# Supplementary material for: Transcriptomic Insights into Mechanisms of Early Seed Maturation in the Garden Pea (Pisum sativum L.)
Source: Cells. 2020 Mar 23;9(3):779. doi: 10.3390/cells9030779 (PMC7140803; doi:10.3390/cells9030779)

Overexpression Spots

landscape

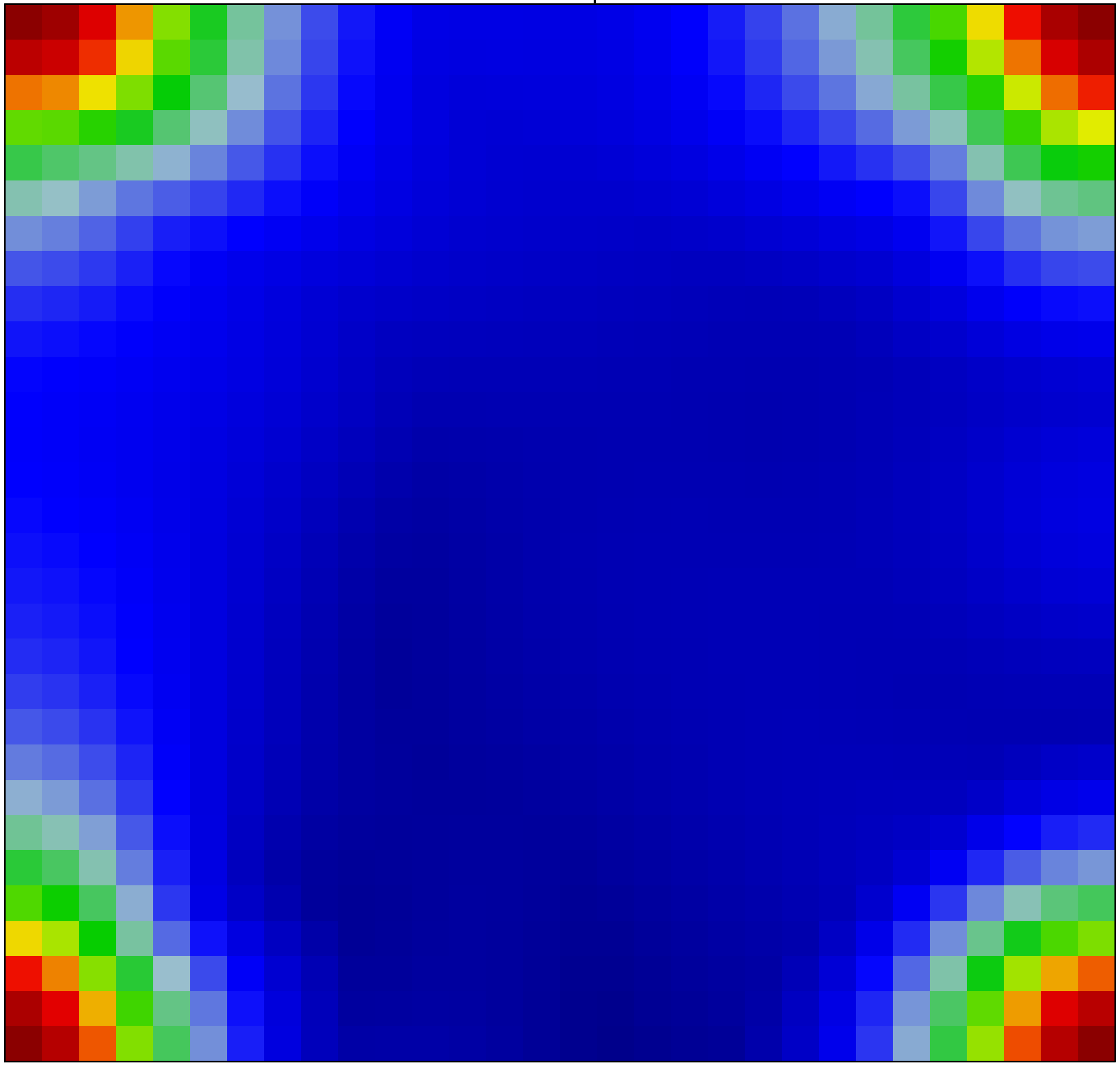

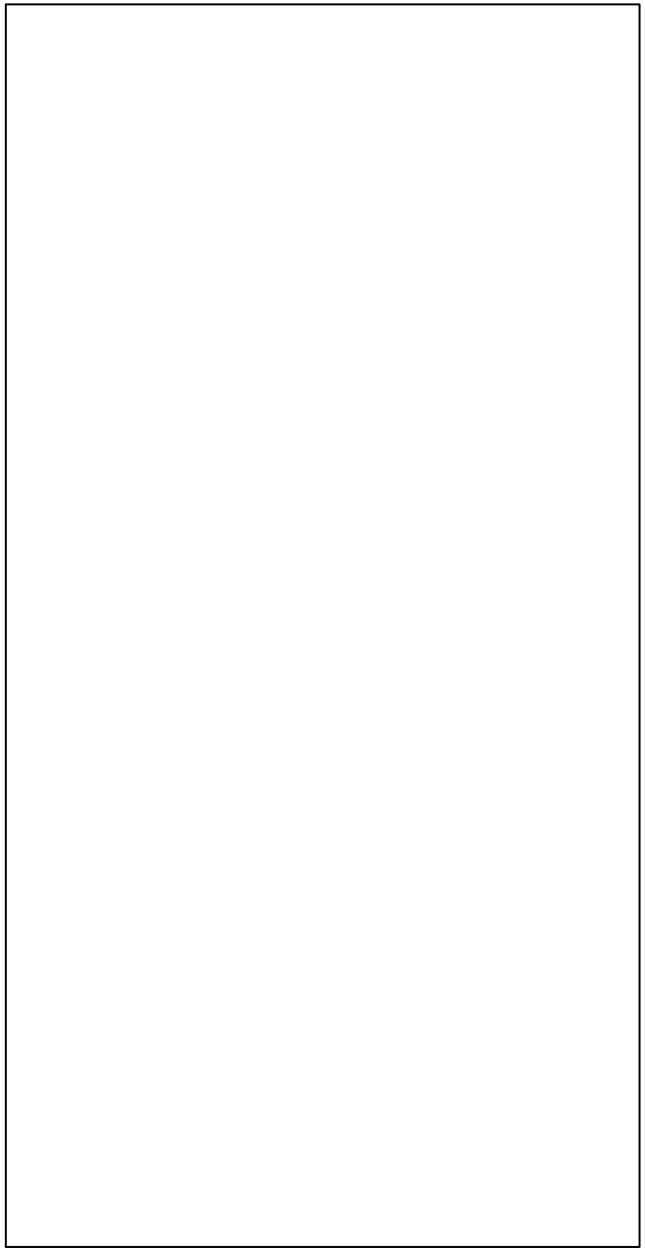

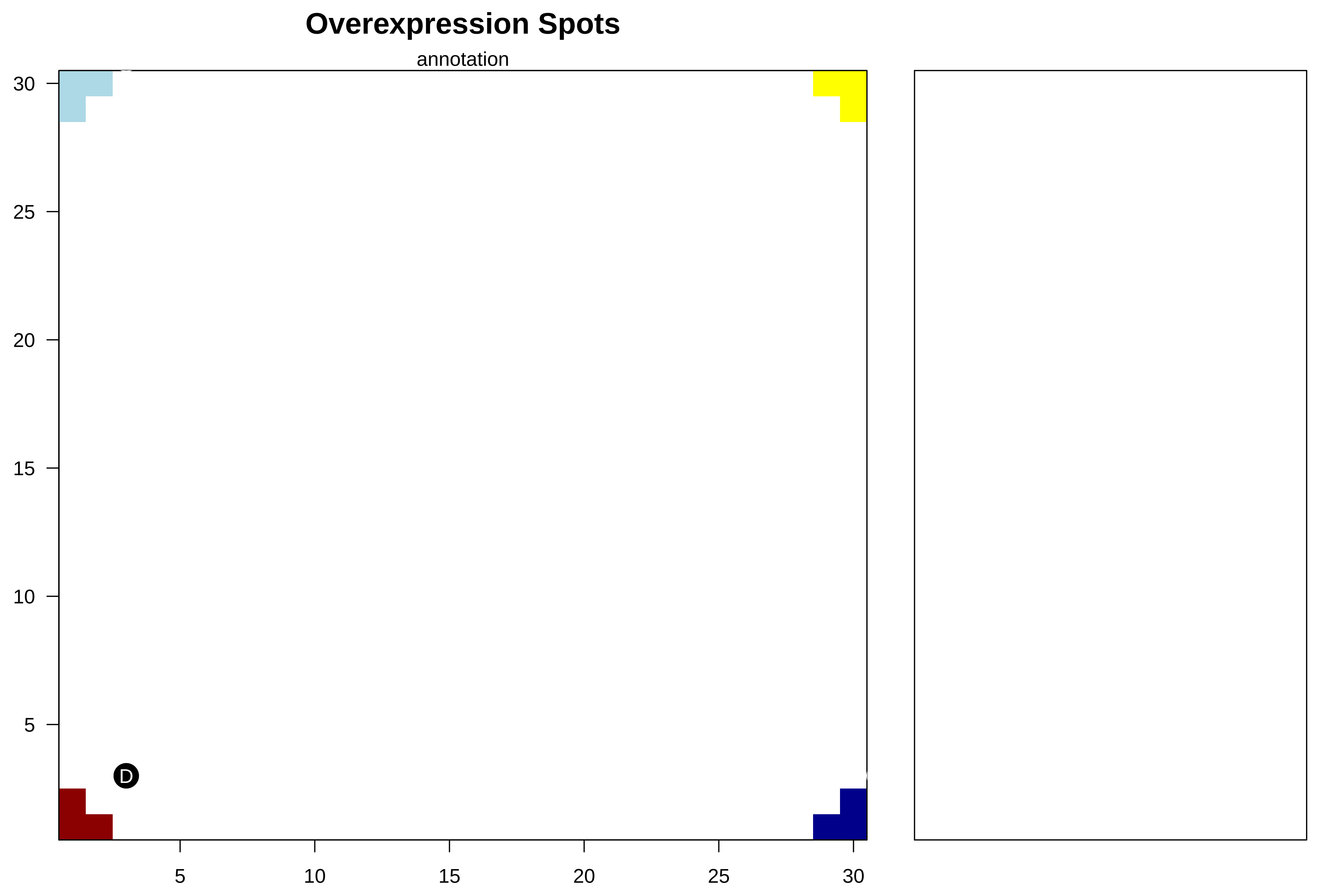
Overexpression Spots
annotation
30
25
20
15
10
5
D
5
10
15
20
25
30

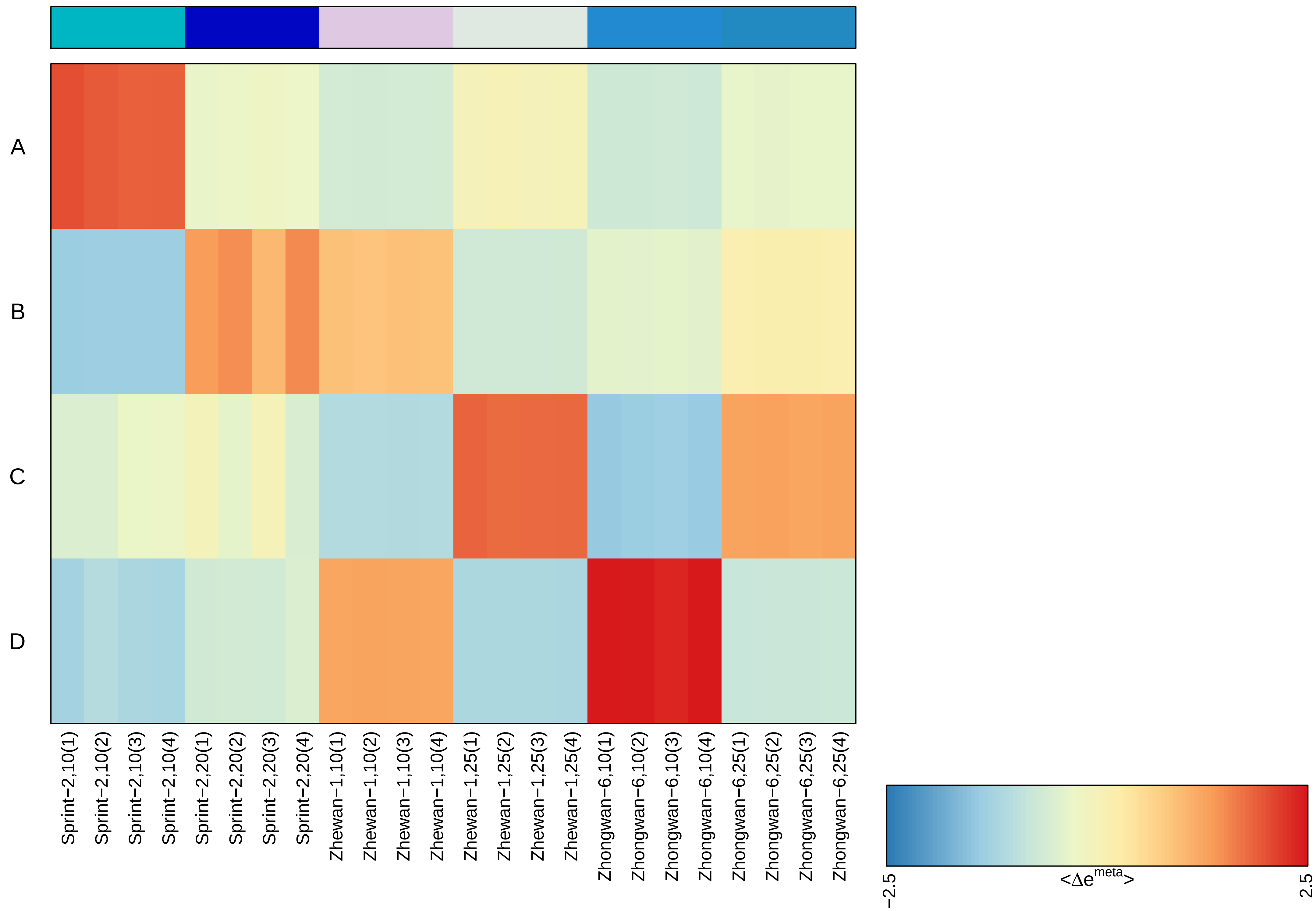
A
B
C
D
Sprint−2,10(1)
Sprint−2,10(2)
Sprint−2,10(3)
Sprint−2,10(4)
Sprint−2,20(1)
Sprint−2,20(2)
Sprint−2,20(3)
Sprint−2,20(4)
Zhewan−1,10(1)
Zhewan−1,10(2)
Zhewan−1,10(3)
Zhewan−1,10(4)
Zhewan−1,25(1)
Zhewan−1,25(2)
Zhewan−1,25(3)
Zhewan−1,25(4)
Zhongwan−6,10(1)
Zhongwan−6,10(2)
Zhongwan−6,10(3)
Zhongwan−6,10(4)
Zhongwan−6,25(1)
Zhongwan−6,25(2)
Zhongwan−6,25(3)
Zhongwan−6,25(4)
−2.5
<Δe^meta>
2.5

Overexpression Spots

Spot Summary: A

metagenes = 3
genes = 58

<r> metagenes = 1
<r> genes = 0.76
beta: r2= 3.88 / log p= −Inf

samples with spot = 4 (16.7 %)
Sprint−2(10) : 4 (100 %)

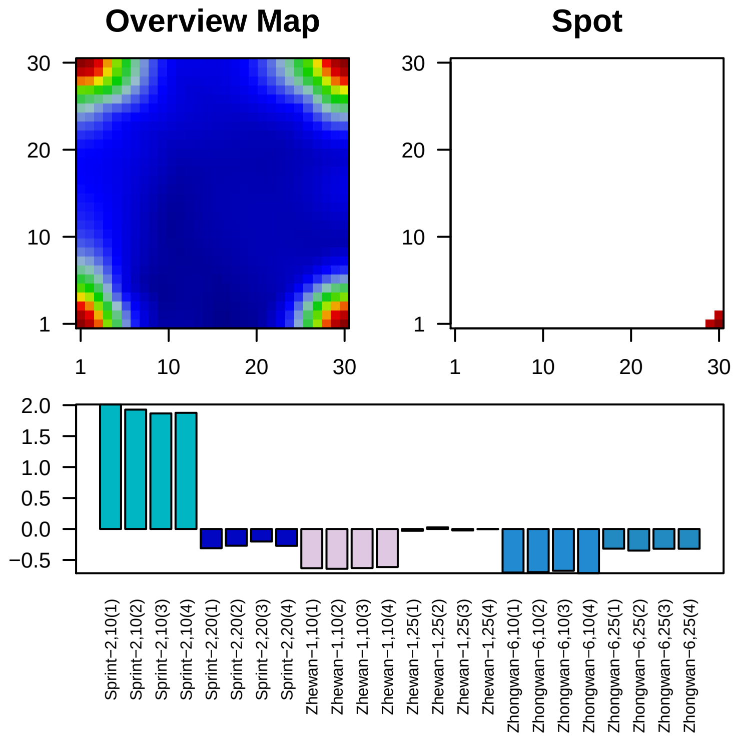

Spot Genelist

Rank	ID	max e	min e	r	Symbol	Description
1	TRINITY_DN5	8.61	−1.68	0.95		
2	TRINITY_DN2	7.6	−8.44	0.84		
3	TRINITY_DN1	7.51	−1.97	0.97		
4	TRINITY_DN1	4.85	−1.19	0.95		
5	TRINITY_DN1	4.67	−2.15	0.92		
6	TRINITY_DN6	4.43	−1.66	0.85		
7	TRINITY_DN9	3.91	−0.84	0.96		
8	TRINITY_DN7	3.84	−2.46	0.45		
9	TRINITY_DN1	3.43	−1.66	0.82		
10	TRINITY_DN1	3.36	−2.28	0.87		
11	TRINITY_DN6	3.29	−1.71	0.92		
12	TRINITY_DN1	3.27	−0.38	0.79		
13	TRINITY_DN7	3	−0.92	0.91		
14	TRINITY_DN2	2.98	−3.37	0.69		
15	TRINITY_DN1	2.98	−1.06	0.98		
16	TRINITY_DN7	2.89	−0.48	0.96		
17	TRINITY_DN3	2.65	−0.56	0.96		
18	TRINITY_DN1	2.6	−0.83	0.97		
19	TRINITY_DN5	2.52	−1.4	0.83		
20	TRINITY_DN5	2.4	−1.02	0.96		

Overexpression Spots

Spot Summary: B

metagenes = 3
genes = 41

<r> metagenes = 1
<r> genes = 0.66
beta: r2= 2.67 / log p= −Inf

samples with spot = 3 (12.5 %)
Sprint−2(20) : 3 (75 %)

Overview Map

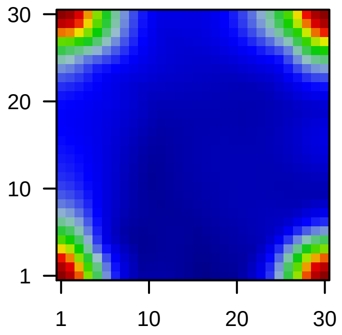

Spot

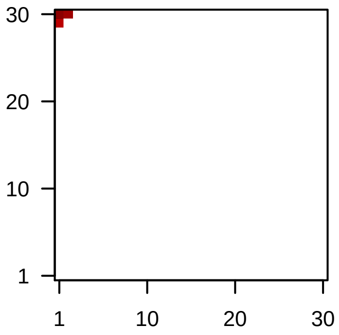

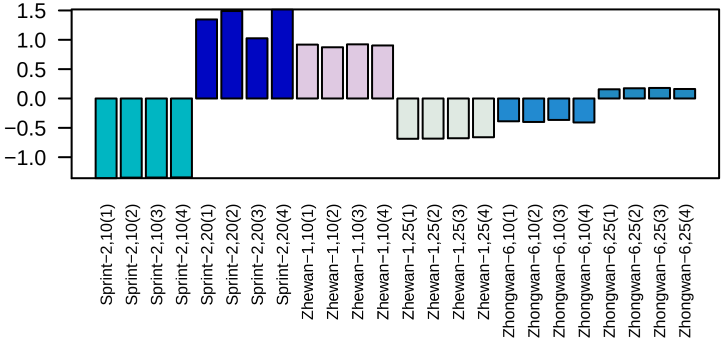

Spot Genelist

Rank	ID	max e	min e	r	Symbol	Description
1	TRINITY_DN4	8.82	−8.66	0.93		
2	TRINITY_DN4	8.39	−5.76	0.93		
3	TRINITY_DN4	5.37	−4.32	0.97		
4	TRINITY_DN1	3.77	−2.56	0.81		
5	TRINITY_DN3	3.65	−1.82	0.74		
6	TRINITY_DN5	3.42	−2.06	0.86		
7	TRINITY_DN4	2.8	−1.11	0.77		
8	TRINITY_DN2	2.61	−1.75	0.8		
9	TRINITY_DN5	2.36	−2.06	0.83		
10	TRINITY_DN5	2.24	−1.8	0.87		
11	TRINITY_DN4	2.18	−1.82	0.81		
12	TRINITY_DN2	2.08	−1.08	0.91		
13	TRINITY_DN1	2.07	−0.94	0.75		
14	TRINITY_DN4	1.85	−1.11	0.75		
15	TRINITY_DN5	1.76	−0.46	0.69		
16	TRINITY_DN1	1.73	−1.06	0.87		
17	TRINITY_DN1	1.71	−1.55	0.65		
18	TRINITY_DN5	1.6	−1.18	0.56		
19	TRINITY_DN1	1.54	−0.9	0.97		
20	TRINITY_DN5	1.53	−0.88	0.87		

Overexpression Spots

Spot Summary: C

metagenes = 3
genes = 45

<r> metagenes = 1
<r> genes = 0.67
beta: r2= 4.95 / log p= −Inf

samples with spot = 8 (33.3 %)
Zhewan−1(25) : 4 (100 %)
Zhongwan−6(25) : 4 (100 %)

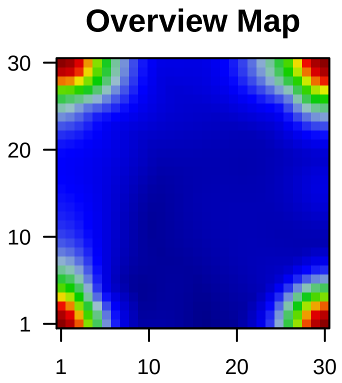

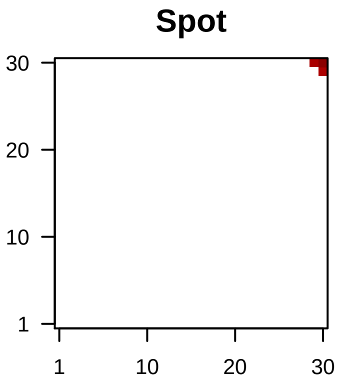

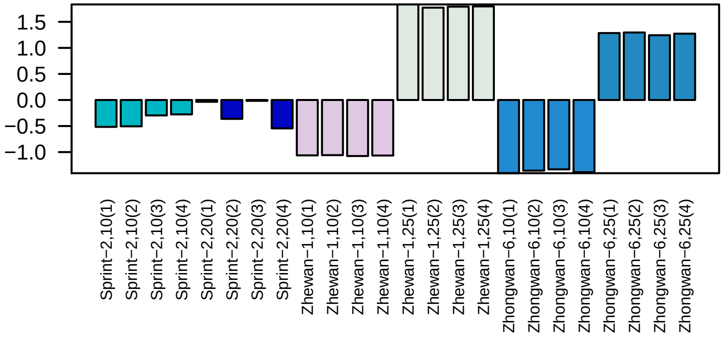

Spot Genelist

Rank	ID	max e	min e	r	Symbol	Description
1	TRINITY_DN1	10.4	−3.02	0.81		
2	TRINITY_DN4	7.91	−7.93	0.94		
3	TRINITY_DN4	7.74	−4.91	0.79		
4	TRINITY_DN1	7.72	−3.91	0.79		
5	TRINITY_DN2	5.9	−1.92	0.78		
6	TRINITY_DN2	4.81	−3.98	0.95		
7	TRINITY_DN5	4.22	−2.39	0.98		
8	TRINITY_DN2	4.11	−3.63	0.9		
9	TRINITY_DN3	3.31	−3.6	0.55		
10	TRINITY_DN1	3.23	−1.54	0.92		
11	TRINITY_DN8	2.99	−2.22	0.84		
12	TRINITY_DN7	2.62	−3.66	0.55		
13	TRINITY_DN6	2.53	−0.84	0.86		
14	TRINITY_DN1	2.27	−1	0.94		
15	TRINITY_DN2	2.25	−1.48	0.57		
16	TRINITY_DN4	2.14	−0.47	0.6		
17	TRINITY_DN1	2.09	−1.85	0.74		
18	TRINITY_DN9	2.04	−0.73	0.84		
19	TRINITY_DN9	1.98	−1.08	0.88		
20	TRINITY_DN4	1.92	−4.32	0.66		

Overexpression Spots

Spot Summary: D

metagenes = 3
genes = 48

<r> metagenes = 1
<r> genes = 0.75
beta: r2= 8.54 / log p= −Inf

samples with spot = 8 (33.3 %)
Zhewan−1(10) : 4 (100 %)
Zhongwan−6(10) : 4 (100 %)

Spot Genelist

Rank	ID	max e	min e	r	Symbol	Description
1	TRINITY_DN1	13.12	−4.78	0.74		
2	TRINITY_DN7	11.07	−3.03	0.95		
3	TRINITY_DN1	8.39	−3.05	0.72		
4	TRINITY_DN1	6.17	−3.24	0.94		
5	TRINITY_DN4	5.77	−4.28	0.73		
6	TRINITY_DN3	5.76	−2.29	0.92		
7	TRINITY_DN1	5.25	−2.61	0.94		
8	TRINITY_DN1	4.57	−1.48	0.97		
9	TRINITY_DN2	4.53	−1.15	0.85		
10	TRINITY_DN3	4.41	−2.6	0.97		
11	TRINITY_DN2	4.05	−1.09	0.8		
12	TRINITY_DN3	3.87	−6.3	0.73		
13	TRINITY_DN3	3.49	−1.03	0.93		
14	TRINITY_DN7	3.48	−1.29	0.99		
15	TRINITY_DN2	3.3	−0.56	0.8		
16	TRINITY_DN2	3.29	−0.85	0.53		
17	TRINITY_DN1	3.26	−3.12	0.74		
18	TRINITY_DN1	2.87	−1.41	0.58		
19	TRINITY_DN2	2.8	−1.04	0.94		
20	TRINITY_DN8	2.78	−0.81	0.9		

Overview Map

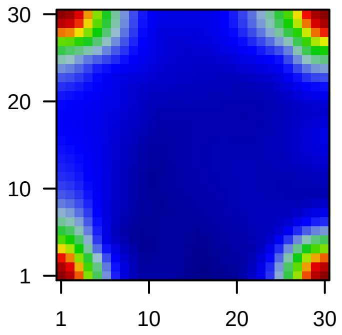

Spot

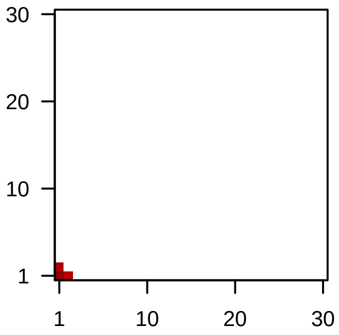

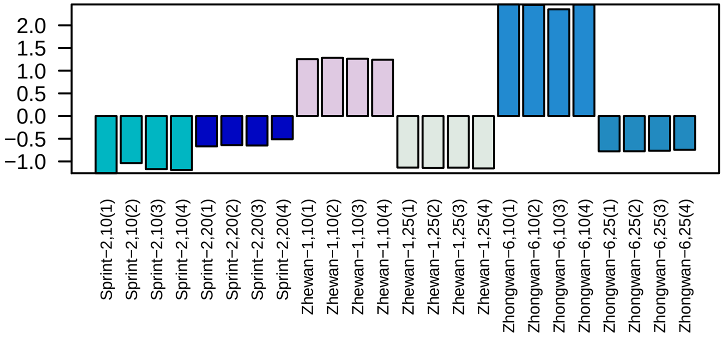

Supplement: Supplementary file 1 [file cells-09-00779-s001.zip › Supplementary materials/FileS4.zip!/FileS3/TFs - Results/Summary Sheets - Modules/Overexpression Spots/Report.pdf]
